# Toxic heavy metal ions contamination in water and their sustainable reduction by eco-friendly methods: isotherms, thermodynamics and kinetics study

**DOI:** 10.1038/s41598-024-58061-3

**Published:** 2024-03-31

**Authors:** Veer Singh, Ghufran Ahmed, Sonali Vedika, Pinki Kumar, Sanjay K. Chaturvedi, Sachchida Nand Rai, Emanuel Vamanu, Ashish Kumar

**Affiliations:** 1 Department of Biochemistry, ICMR-Rajendra Memorial Research Institute of Medical Sciences, Patna, 800007 India; 2 Department of Microbiology, ICMR-Rajendra Memorial Research Institute of Medical Sciences, Patna, 800007 India; 3 Centre of Experimental Medicine and Surgery, Institute of Medical Sciences, Banaras Hindu University, Varanasi, 221005 India; 4 Faculty of Biotechnology, University of Agricultural Sciences and Veterinary Medicine of Bucharest, 011464 Bucharest, Romania

**Keywords:** Heavy metals, Water contamination, Biosorption, Eco-friendly biosorbent, Biotechnology, Environmental sciences

## Abstract

Heavy metal ions can be introduced into the water through several point and non-point sources including leather industry, coal mining, agriculture activity and domestic waste. Regrettably, these toxic heavy metals may pose a threat to both humans and animals, particularly when they infiltrate water and soil. Heavy metal poisoning can lead to many health complications, such as liver and renal dysfunction, dermatological difficulties, and potentially even malignancies. To mitigate the risk of heavy metal ion exposure to humans and animals, it is imperative to extract them from places that have been polluted. Several conventional methods such as ion exchange, reverse osmosis, ultrafiltration, membrane filtration and chemical precipitation have been used for the removal of heavy metal ions. However, these methods have high operation costs and generate secondary pollutants during water treatment. Biosorption is an alternative approach to eliminating heavy metals from water that involves employing eco-friendly and cost-effective biomass. This review is focused on the heavy metal ions contamination in the water, biosorption methods for heavy metal removal and mathematical modeling to explain the behaviour of heavy metal adsorption. This review can be helpful to the researchers to design wastewater treatment plants for sustainable wastewater treatment.

## Introduction

The issue of heavy metal pollution is increasingly pervasive on a global scale. Heavy metals are naturally occurring elements that are present in the earth's crust. However, excessive amounts of heavy metals can pose a significant risk^1^. Some compounds, such as heavy metals, are resistant to decomposition and can accumulate in people and animals when they enter the food chain^2^. Metals can enter the environment through natural means or human actions including waste disposal, industrial manufacturing, and mining^3^. Mining poses a significant danger by potentially displacing and spreading heavy metals to surrounding regions during flooding or windstorms^4^. It is important to acknowledge and address environmental hazards to safeguard the well-being of both humans and the natural world^5^.

Heavy metal-induced water pollution can have detrimental impacts on human health^6,7^. These metals can enter our systems via polluted water and food^8^. They can bind with organic groups, resulting in the formation of detrimental chemicals that can induce damaging effects on our cells^9^. Multiple techniques exist for extracting these metals from polluted water; however, they are accompanied by drawbacks such as the production of additional pollutants or exorbitant expenses^10–13^. Hence, it is crucial to devise appropriate biological techniques for the remediation of heavy metals^14^.

Biosorption is an efficient and eco-friendly technology created to remove heavy metal ions from polluted water, offering both cost-effectiveness and environmental benefits^15^. Biosorption methods can replace conventional methods and can be considered as suitable alternative to existing physiochemical methods due to the eco-friendly and cost-effective nature of biosorption techniques. The biosorption method relies on the utilization of various types of raw materials derived from agro-waste, plant residue, and algal and microbial biomass^16^. Biosorption is a metabolically independent method that does not require the participation of living organisms, making it a more straightforward and user-friendly technology^17^. A diverse range of materials, such as rice and wheat husks, activated carbon, agricultural waste, bananas and citrus peels, and green-synthesized nanoparticles, can be effectively used for biosorption^18–20^. It is important to emphasise that these materials have a distinct surface character that greatly enhances their capacity to absorb the heavy metal ions found in the water^21,22^.

This review is focused on the heavy metals contamination sources including point and non-point sources of heavy metal ions contamination in the water. This review also provides detailed information on the biosorption method for heavy metal removal. In addition, the behaviour of biosorption is also described by mathematical models including isotherms, thermodynamics and kinetics.

## Water quality assessment

### Water quality criteria

A thorough examination of a wide range of variables that are well-known and recognized as key indicators to accurately characterize the overall quality of water^23^. The World Health Organization recommends the maximum allowable limits for water physicochemical parameters, as shown in Table 1.

**Table 1:** The assessment of water quality parameters and their corresponding allowable thresholds in potable water sources (source: https://www.who.int/water_sanitation_health/dwq/fulltext.pdf).

Parameters	Permissible limits
pH	6–9
Temperature	25
Total solids (mg/l)	1500
Nitrate (mg/l)	50
Ammonia (mg/l)	1.5
Ni (mg/l)	0.07
Zn (mg/l)	0.05
Cd (mg/l)	0.003
Pb (mg/l)	0.01
Ti (mg/l)	0.05
Cr (mg/l)	0.05
As (mg/l)	0.01

This comprehensive understanding and assessment of the various variables are based on the findings and conclusions drawn from a meticulous study conducted by several researchers^24^. To ensure the utmost safety and well-being of users who rely on water, whether it is for consumption, recreational activities, or any other specific purpose, several essential water quality criteria are implemented and enforced. These criteria are meticulously designed to regulate and control the maximum allowable concentration level of specific substances within a given medium, be it water, sediment, or biota. The primary objective behind these criteria is to prevent and eliminate any potential risks or harmful effects that may arise from exposure to excessive levels of such substances. It is important to note that these water quality criteria are particularly crucial and significant when the medium, such as water, sediment, or biota, is continuously utilized or relied upon for a specific purpose. This emphasis on continuous usage further highlights the necessity and importance of establishing and adhering to these criteria to ensure long-term safety and sustainability.

It is imperative to acknowledge and recognize the multifaceted nature and complexity of these physicochemical parameters, as they collectively play a pivotal role in determining and shaping the overall quality and characteristics of water. Their interconnected and interdependent nature necessitates a thorough understanding of each parameter's influence and impact on water quality. Moreover, the presence and concentration of heavy metals in water are of particular concern and importance due to their potential toxicity and detrimental effects on both human health and the environment^25^. The establishment and enforcement of water quality criteria, alongside regular monitoring and assessment of heavy metals concentration, are crucial in safeguarding and preserving the integrity and safety of water resources^26^. The water quality criteria are vital components of ensuring the safety, sustainability, and overall well-being of users who rely on water for various purposes. These criteria are meticulously formulated based on a detailed understanding and examination of numerous variables that accurately characterize water quality. The comprehensive assessment of physicochemical parameters, including dissolved oxygen levels, pH, temperature, conductivity, BOD, COD, TDS, minerals, and heavy metals concentration, is essential in maintaining and protecting the quality and integrity of water resources^27^.

Following the comprehensive and exhaustive evaluation and analysis of the ambient water quality concerning the implementation and execution of appropriate and effective measures and actions to adequately and proficiently manage and control pollution for all types of discharges, including those occurring in the upstream sections of the water bodies^28^. It is significant to acknowledge and recognize that this particular mechanism and approach also serves and functions as a means and tool to facilitate and support the growth, development, and establishment of various industries, thereby emphasizing and emphasizing the crucial and pivotal significance and role it plays in the overall and comprehensive framework and structure of environmental management. It is imperative and essential to explicitly state and specify that under no circumstances and situations are industries permitted or authorized to release or discharge any form or type of waste or effluent materials into the river sections^28,29^.

### Water quality assessment and management

There is a problem with water quality around the world. The preservation of public health, food security, biodiversity, and additional ecosystem services are progressively endangered by the intensifying and escalating pollution of fresh water in both developed and developing nations^30–33^. A noteworthy association exists between pollution and economic advancement, with population growth, agricultural expansion, industrial expansion, and energy production all contributing to the discharge of untreated or uncontrolled wastewater into surface and groundwater bodies. Despite recent preliminary evaluations of water quality worldwide, the extent of the predicament remains uncertain^34^. Water quality needs to be protected and improved effectively and efficiently with better information about the issues involved. Government and private agencies are working on water quality assessment and management^35^.

1. The development and implementation of a comprehensive water resources plan, policy formulation, coordination, and guidance.
2. Irrigation, flood control, and multi-purpose projects need to be closely monitored, supervised, inspected, cleaned, and monitored for their effectiveness.
3. Groundwater development is the process of developing groundwater resources, establishing utilizable resources, and formulating policies for their exploitation, along with the supervision of state-level groundwater development activities and the support that is provided to them.
4. The development of a comprehensive perspective regarding the water resources of a nation and the assessment of the water balance across various basins and sub-basins are key considerations in the evaluation of inter-basin transfer feasibility.

The primary initiatives that are currently being undertaken involve a comprehensive investigation into the management of groundwater, both at macro and micro levels. These measures play a crucial role in ensuring the sustainable management of groundwater resources. It is of paramount importance to prioritize these initiatives to guarantee the long-term viability of groundwater resources^36^. Furthermore, the Board, in collaboration with concerned state government agencies, conducts periodic evaluations of replenishable groundwater resources in the country. This collaborative approach ensures a comprehensive and informed understanding of the current state of groundwater resources^37^.

The Central Pollution Control Board (CPCB) of India and the Environmental Protection Agency (USA) are authoritative bodies, that exercise their oversight over the numerous state boards by setting emission standards and establishing ambient standards^38^. These bodies play a crucial role in mitigating the adverse effects of pollution by conducting nationwide surveys to evaluate the existing state of pollution. To achieve this goal, the Environmental Protection Agency has implemented two comprehensive monitoring programs for inland water quality. Through these programs, a network of 480 measurement stations such as tanneries, chemical plants, textile mills and distilleries has been established across the primary river basins in the country^39^. These measurement stations serve as crucial points of data collection and analysis, enabling a comprehensive understanding of water quality^40–43^.

Moreover, it is essential to recognize the significance of the field of International Environmental Law (IEL) in safeguarding our planet's environment, which is a shared resource. At AIDA, it is necessary to actively engage with this field daily, utilizing its principles and frameworks to support individuals and communities in their efforts to protect the environment. Preserving the environment is closely intertwined with the protection of foundational human rights. Therefore, our work in the field of IEL not only seeks to safeguard the environment but also aims to uphold and promote these fundamental rights that are inextricably linked to the environment. Through our commitment to the principles and practices of IEL, to strive to contribute to a sustainable and equitable future for all^44^.

## Occurrence of heavy metals in the environment

For each 10% increase in the usage of pesticides, this phenomenon can be observed. Many investigations have been carried out on the subject of wastewater and its impact on human health. A study examining the influence of irrigation water quality on human health discovered higher rates of illness in the villages that employed wastewater for irrigation in comparison to the control village^45^. A study conducted by Bartone^46^ observed that water pollution serves as both a cause and an effect in the connections between agriculture and human health^46^. The contamination of heavy metals in water is also influenced by natural factors such as volcanic activity, metal corrosion, metal evaporation from soil and water, soil erosion, and geological weathering^47^. In comparison to the global average level, the concentration of trace elements (> 0.05 mg/L hexavalent chromium and > 0.01 mg/L arsenic) in water quality on the Child Loess Plateau is found to be higher. The quality of river water, when poor, is associated with high levels of sodium and salinity hazards. In the case of surface water bodies, a wide range of pollution sources, including both point and non-point sources, have a significant impact on water quality^48^.

### Point source

Point sources of toxic heavy metal ions contamination in water are defined as particular types of pollution that cause high amounts of heavy metal ions contamination in water. It is important to release contaminants from the sources and directly inject them into the nearest water bodies or environmental sources^49^. Industrial units situated on the banks of the rivers serve as major heavy metal contamination sources in the water. The point sources of heavy metals contamination are described into two main categories^49^.

#### Industrial sources

Industrial wastewater plays a major role in the heavy metals contamination in the water. Industrial wastewater contains several hazardous chemicals including heavy metal ions that are directly or indirectly released into the environment. These heavy metal ions accumulate in the food chain and affect human beings including terrestrial and aquatic animals^50^. Based on a survey by the central pollution control board, 260 million litres of industrial wastewater daily released into the Ganga River in India^51^. According to a report released by the Ministry of Ecology and Environment of China, the nation released a total of 25.02 billion tons of industrial wastewater in the year 2019, which is equivalent to approximately 68.5 billion litres per day^52^. As stated in a report published by the World Bank, industries operating in Bangladesh discharge an approximate amount of 1.5 million cubic meters (equivalent to 1.5 billion litres) of untreated or partially treated wastewater into rivers and other water bodies daily^53^. There are several industries which cause heavy metal contamination in water paper, sugar, textiles, steel, battery, leather, chemicals, pharmaceuticals, metal works, and food industries discharge their wastewater into the environment^54,55^.

#### Domestic sources of pollution

The domestic source of water contamination is the second major part of a point source. Domestic sources also depend on the collection of waste and their dumping^56,57^. Domestic pollution can be reduced if wastewater is properly treated before discharging into the environment^58^. The main components of domestic sources are microbes and organic matter. Domestic waste also contains a large amount of metals and salt including chlorides, detergents, oils and grease. The Yamuna River in India is highly polluted by domestic sources, about 85% of the other sources of contamination^59^. In China, the sources of water contamination from within the country comprise industrial emissions, untresated domestic sewage, and agricultural overflow. Based on current information, industrial wastewater is a significant contributor to water pollution, as more than 60% of China's underground water and a third of its surface water are classified as unsuitable for human use due to contamination^60^. In Bangladesh, various factors like insufficient sewage treatment, industrial wastage, and agricultural runoff contribute to water pollution. Studies suggest that a significant percentage of surface water in Bangladesh, around 85%, is contaminated. This contamination mainly stems from domestic and industrial sources, resulting in severe health problems for millions of people who depend on polluted water sources^53^.

### Nonpoint or diffused source of pollution

The contributions originating from sources that are spread out and not concentrated in one specific location are deemed to be of lesser significance when compared to the contributions from sources that are concentrated in one specific location. This is primarily because these diffused sources lack specificity in terms of their characteristics and attributes, and also due to their sheer abundance in number^61^. When pollutants, such as harmful substances or contaminants, are discharged and flow into a body of water, they are categorized as nonpoint sources. These nonpoint sources can arise from various activities or areas, without a specific source or location to attribute them to. For instance, runoff from a field has the potential to carry fertilizers and pesticides into a nearby stream, thereby contributing to the pollution of the water. The fertilizers and pesticides used in agriculture contain several types of metal compounds^62^. These metals cause several types of contamination in the water. The occurrence of monsoon, which is a period characterized by heavy rainfall, plays a significant role in the process of leaching, drainage, and surface water runoff^63,64^. These processes serve as mechanisms or pathways through which pollution is transported from the catchment area of a river to the river itself. It is important to note that the pollution being transported in this manner is predominantly diffused in nature, meaning that it is made up of various components that are not concentrated in one specific location. These components include but are not limited to topsoil, organic matter, plant residues, nutrients, toxicants, and microorganisms. Thus, the diffused pollution being transported in this manner encompasses a diverse range of substances and materials^62,64^.

#### Agricultural sources of pollution

The pollution of rivers caused by agricultural activities is linked to a variety of crucial elements, specifically, the remnants left behind from agricultural practices, the utilization of fertilizers and pesticides, the rearing of livestock, and the excessive accumulation of salts that arise as a consequence of the implementation of irrigation water^65^. The waste generated from agricultural activities within the watershed of the river undergoes a natural process of decomposition, ultimately culminating in the contamination of the river^66^. The agricultural residues are also part of the food chain specially utilized by bacteria and fungi. Their microorganisms break down agricultural waste and these degraded waste materials are responsible for water contamination^67^.

### Landfill and dumping of toxic waste

The dumping and landfill of hazardous materials are done carefully and follow the guidelines of CPCB, India and other environmental protection agencies. The scope of Municipal Solid Waste (MSW) is wide-ranging, encompassing not only household waste but also healthcare and industrial refuse^68^. However, it is concerning that there is a lack of adequate categorization for these different types of waste, resulting in their indiscriminate deposition into a single landfill. This indiscriminate disposal practice has significant consequences for the environment and public health, as it leads to severe pollution of both the immediate and surrounding areas^69^. The landfill, being the primary location for the disposal of solid waste, plays a central role in these detrimental effects. The repercussions of such disposal methods are far-reaching, with environmental pollution and the spread of diseases being particularly severe outcomes^70^. A specific concern in the context of open dumping sites is the transportation of leachate, which serves as a prominent source of heavy metals in various environmental compartments such as surface and groundwater, soil, and vegetation^71^. The heavy metals that are of particular concern in this regard include Cd, Cr, Cu, Pb, Ni, and Zn, as they pose significant issues due to their presence and potential for harm^72^. Furthermore, the impurity of wastewater has emerged as a direct cause of the contamination of food crops, further exacerbating the overall issue at hand^73^.

#### Other sources of water pollution

There exist additional origins of water contamination, including but not limited to the excessive utilization of water for bathing and clothes washing, the practice of cattle wading, and the act of open defecation^74^. Bathing and cloth washing in the river are among the most prevalent activities that are closely associated with water pollution. In various towns and villages located along rivers, it is customary to lead cattle to the river for drinking and bathing^75^. The impact of cattle-related activities on water quality cannot be underestimated. This is evident through the direct release of urine, dung, and both organic and inorganic matter that gets washed off from the cattle. These activities not only contaminate the water but also have a significant influence on its overall quality. Moreover, when cattle wade through rivers, they disturb the sediments present at the riverbed, further exacerbating the situation by introducing additional pollutants into the water^76^. It is important to note that these issues are not limited to rural areas alone. Even in urban areas, especially in slum clusters where proper sanitation facilities are lacking, open defecation is rampant. This leads to a surplus of waste being dumped into open spaces. Consequently, a considerable portion of the population resorts to using either the catchment area or the river itself as a means of waste disposal. This further contributes to the introduction of organic pollution and pathogens into the river water, exacerbating the already compromised quality^77–80^.

## Biosorption of heavy metal ions

**Figure 1:**
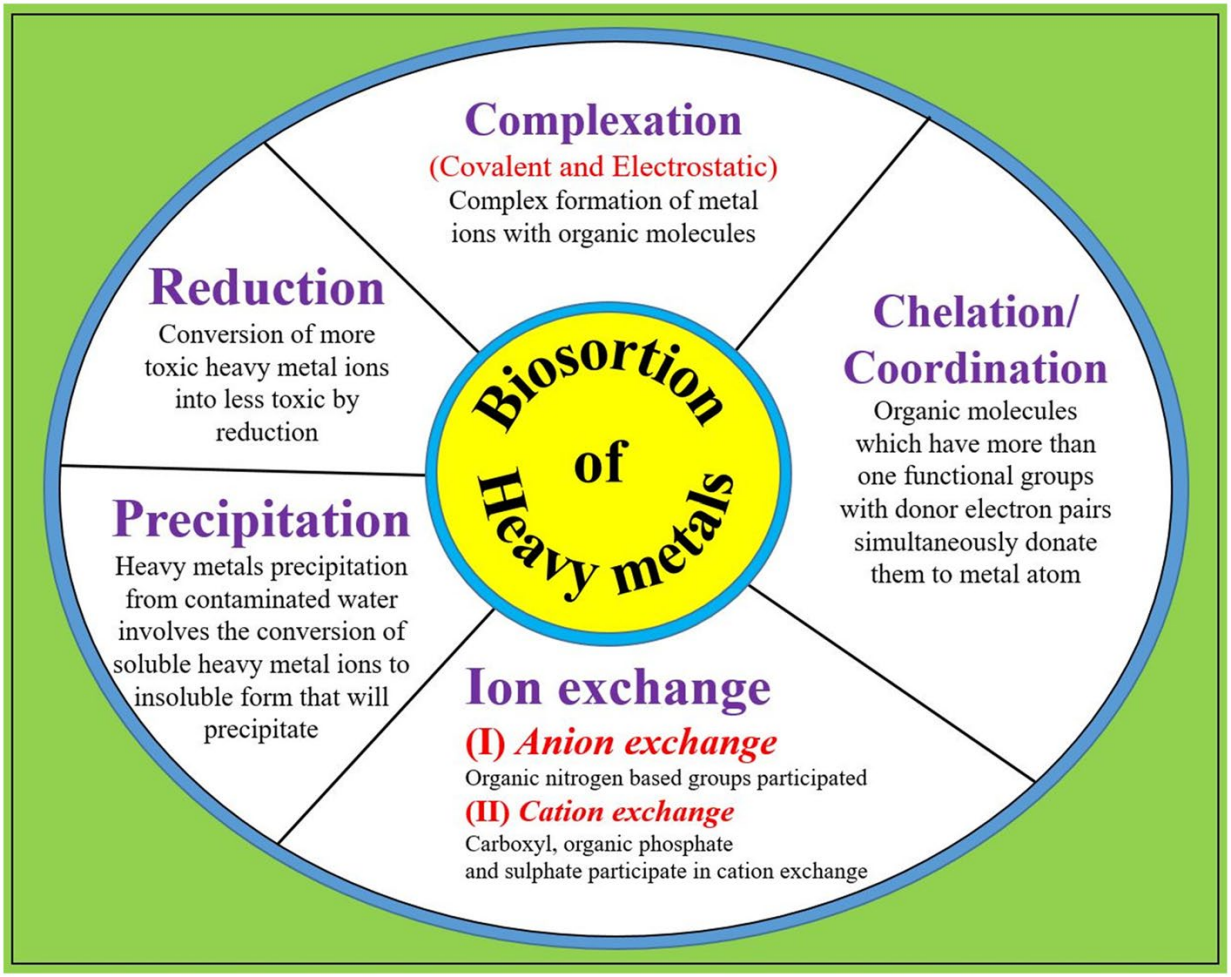
Several processes involved in the biosorption of heavy metal ions.

There exist various methodologies by which wastewater may be cleansed of hazardous compounds, including but not limited to heavy metals. One such technique, referred to as biosorption, involves the utilization of expired microbial biomass for the express purpose of extracting these aforementioned metals^81^. This particular approach is further elucidated and visually demonstrated within a schematic representation, as denoted by Fig. 1.

It is widely believed that dead plant material can be used to remove heavy metals from polluted water. This process, called biosorption, happens when the metal ions stick to the surface of the dead plant material^82^. Interestingly, living plants can also do this. In living plants, the metal ions can stick to the surface of the cells or get absorbed through the plant's metabolic processes. This is an important process to help clean up polluted water^83^.

In the process of removing harmful heavy metal ions from water, a natural and cost-effective solution is to use biosorbents^83^. These are materials that contain certain functional groups, such as amino, amide, imidazole, sulfonate, and carboxyl groups, that can bond with heavy metal ions to remove them from water^84^. The effectiveness of biosorbents depends on the variety and concentration of functional groups present on their surface, as well as their surface shape^85^. The rough and porous surface of biosorbents is better at binding heavy metal ions and removing them from water^86^. Scientists use various methods such as FT-IR, SEM, EDX, NMR, and XRD to analyze the surface shape and functional groups of biosorbents and ensure their quality^87^.

The process of biosorption can be influenced by numerous factors, including the utilization of specific microorganisms, the existence of various metal ions (including those that contend with the target metal), temperature, and pH^88,89^. If the pH level decreases, the competition among metal ions that possess a positive charge can intensify^89^. Conversely, if the pH level rises, a greater number of surface binding sites become accessible. The mechanism of biosorption for hexavalent chromium ions (Cr (VI)) is rather intricate^90^. These ions possess the ability to adhere to groups on the surface that are positively charged and subsequently undergo a transformation into trivalent chromium ions (Cr (III)) via diverse pathways. Ordinarily, this process transpires in three distinct stages^91^.

The initial step in the biosorption process involves the attachment of positively charged surface functional groups to negatively charged Cr (VI) ions. The subsequent stage of the biosorption process is the reduction process. The conversion of Cr (VI) to Cr (III) is facilitated by electron donor groups^17,92,93^. Heavy metals biosorption capacity of different adsorbents are mentioned in the Table 2, 3, 4 and 5.

**Table 2:** Cr (VI) biosorption capacity of different biosorbents.

Biosorbent	Biosorption capacity (mg/g)	pH	Temperature (°C)	References
*Bacillus salmalaya*139SI	20.35	3	25	^94^
*Opuntia* biomass	18.5	2	20	^95^
*Opuntia* biomass	16.5	2	20	^96^
*Dictyota dichotoma* biomass	9.02	4	27	^97^

**Table 3:** Cd (II) biosorption capacity of different biosorbents.

Biosorbent	Biosorption capacity (mg/g)	pH	Temperature (°C)	References
Okara waste	14.80	6.2	70	^98^
Maize corncob	105.6	6	**–**	^99^
Sugarcane bagasse	69.06	6	**–**	^99^
Wheat straw biochar	69.80	5	25	^100^
*Klebsiella* sp. biomass	170.4	5	30	^100^
Alga *Anabaena sphaerica* biomass	111.1	5.5	25	^100^

**Table 4:** Pb (II) biosorption capacity of different biosorbents.

Biosorbent	Biosorption capacity (mg/g)	pH	Temperature (°C)	References
*Citrus grandis* peels	2.13	3	50	^101^
Pea (*Pisum sativum*) peels	140.84	6	30	^102^
Gingelly oil cake (thermally activated)	105.26	**–**	**–**	^103^
Meranti sawdust	34.24	6	30	^104^
*Solanum melongena* leaves	71.42	5	40	^105^
*Araucaria heterophylla* (green plant) biomass	9.64	5	30	^106^
*Azadirachta indica* A. Juss seeds	17.96	5.5	–	^107^

**Table 5:** As (III/V) biosorption capacity of different biosorbents.

Biosorbent	Biosorption capacity (mg/g)	pH	Temperature (°C)	References
Watermelon peel waste	0.00242	5.5–7.5	–	^108^
*Moringa oleifera* seeds	99.9%	4	–	^109^
Chemically modified *Chlorella vulgaris* biomass	20.9	6	25	^110^
Chemically modified *Spirulina platensis* biomass	24.8	6	25	^111^
Dried microalga *Chlamydomonas* sp. biomass	53.8	4	25	^112^

## Modelling approaches for heavy metals biosorption

### Isotherm models

Isotherms, which are indispensable tools in the realm of adsorption investigations, are primarily concerned with the meticulous and intricate analysis of the multifaceted and convoluted correlation existing between the adsorption capacity of a given substance and the residual concentration of heavy metal ions that are inherently present therein, all while ensuring that the temperature remains constant. In the vast field of adsorption, an abundance of isotherm models has been introduced and extensively employed, encompassing, yet not limited to, the renowned Freundlich, Langmuir, Temkin, Halsey, Harkin-Jura (H-J), D-R, Redlich-Peterson, and Jovanovic isotherm models, all of which possess their own distinct merits and drawbacks when it comes to the accurate prediction of adsorption behaviour^113^.

#### Langmuir isotherm

The fundamental principle that forms the basis for the Langmuir isotherm is founded on the concept of monolayer adsorption, which takes place exclusively on a homogenous adsorbent. This phenomenon is achieved by disregarding any potential surface interaction that may occur between two molecules that have been absorbed into the adsorbent material^114^. The mathematical expression is shown in Eq. (1).

1 $$\frac{{C}_{e}}{{q}_{e}} = \frac{1}{{Q}^{0}b} + \frac{{C}_{e}}{{Q}^{0}}$$

Recent investigations have employed the Langmuir isotherm to explore adsorption phenomena in diverse domains, including environmental remediation^115,116^.

#### Freundlich isotherm

In stark contrast, the model known as the Freundlich adsorption isotherm delves into the intricate realm of multilayer adsorption occurring on the surface of an adsorbent that is characterized by its heterogeneity. This particular model serves the purpose of elucidating the underlying mechanisms involved in the process of adsorption, which is fundamentally centred around the deposition of numerous layers of molecules onto the surface of said adsorbent. This elaborate process is achieved through a meticulous examination of the heterogeneity displayed by the surface of the adsorbent, as well as a thorough analysis of the intricate interactions that transpire between the absorbing molecules and the material constituting the adsorbent^117^.

The linear form of Freundlich isotherm is given in Eq. (2).

2 $${\mathrm{log }\, q}_{e} =\mathrm\, { log }kf + \frac{1}{n}\mathrm{log }\, {C}_{e}$$

The investigation of heavy metal adsorption processes, especially in environmental remediation, has been the focus of recent studies that have utilized the Freundlich isotherm^115,118^.

#### Temkin isotherm

The Temkin isotherm model offers a prognostication of equivalent binding energies for the adsorption on surfaces, thereby enabling a more comprehensive comprehension of the process. It has been noted that the heat associated with adsorption rises linearly alongside the number of binding sites within a given layer. This implies that the adsorption process is predominantly influenced by the even dissemination of binding energies, albeit only until a certain threshold, for all molecules present within said layer^119^. The Temkin isotherm is shown in Eq. (3).

3 $$q_{e} = \frac{RT}{{b_{T} }}{\text{ln\, A}}_{{\text{T}}} + \frac{RT}{{b_{T} }}{\text{ln C}}_{{\text{e}}}$$

Recent investigations have recently employed the Temkin isotherm, a widely utilized mathematical model, to comprehensively examine the intricate mechanisms underlying heavy metal adsorption processes, with a specific focus on their application in the realm of environmental remediation, as explicated by the works of Nguyen et al.^115^ and Raji et al.^120^.

### Dubinin–Radushkevich (D–R) isotherm

The D–R isotherm model posits that the adsorption process of heavy metal ions is deeply contingent upon the intricate and nuanced characteristics intrinsic to the structure of the adsorbent material^121^. The linear form of D–R isotherm is shown in Eq. (4).

4 $${\text{ln}}\, q_{e} = {\text{ ln Q}}_{{\text{D - R}}} - \, \beta \, \varepsilon^{2}$$

where, Q_D–R_ (mol/g) and ꞵ (mol^2^ kJ^−2^) are the D–R constants, calculated from the intercept and slope of the plot between ln qe and ɛ^2^. Here, ɛ is Polanyi potential and is calculated from Eq. (5).

5 $${\upvarepsilon }^{2}=RT\mathrm{ ln}\, \left(1+\frac{1}{{C}_{e}}\right)$$

where R is the universal gas constant (8.341 J mol^−1^ K^−1^) and T is the temperature (K).

The relationship between the free energy of adsorption and the D–R isotherm constant is established. The free energy signifies the amount of energy required for the adsorption of one mole of adsorbate. It is possible to determine this value by utilizing Eq. (6).

6 $$E=\frac{1}{\sqrt{-2\beta }}$$

where E (kJ mol^−1^) is the free energy which denotes whether the adsorption system is physical or chemical.

Recent research has employed the D–R isotherm, referred to as the Dubinin-Radushkevich isotherm, as a valuable tool in investigating and exploring the mechanisms and processes of heavy metal adsorption, especially in the context of environmental remediation efforts^115,122^.

#### Halsey isotherm

On the contrary, the Halsey isotherm model delineates the phenomenon of multilayer adsorption occurring at a significantly greater spatial separation from the surface of the adsorbent^123^. Equation (7) exhibits the Halsey isotherm.

7 $${q}_{e}=\frac{1}{{n}_{H}}{I}_{n}{K}_{H}-\frac{1}{{n}_{H}}{\text{ln}}\, {C}_{qe}$$

Recent studies have applied the Halsey isotherm to investigate heavy metal adsorption processes, especially in environmental remediation^124^.

### Harkin–Jura (H–J) isotherm

The Harkin–Jura (H–J) isotherm model talks about how adsorbents (materials used to remove pollutants from liquids or gases) can have multiple layers of pollutants sticking to their surface^125^. H–J isotherm is shown in Eq. (8).

8 $$\frac{1}{{q}_{e}^{2}}= \frac{B}{A}- \left(\frac{1}{A}\right)log\, {C}_{e}$$

where B and A are the model constants. B and A can be calculated from the slope and intercept of the plot between $\frac{1}{{q}_{e}^{2}}$ versus $log{ C}_{e}$.

Liosis et al.^126^ and Czikkely et al.^127^ described H–R isotherm modeling in their study for heavy metal adsorption processes, especially in environmental remediation.

### Thermodynamics

To put it simply, we can study how certain materials interact with each other under different temperatures. If we see a positive change in one property called enthalpy (∆H°), it means that the process needs more heat to happen and we can make it happen faster by increasing the temperature. On the other hand, if we see a negative change in another property called Gibbs free energy (∆G°), it means that the process can happen on its own and will happen faster if we increase the temperature^128^. Thermodynamic parameters were calculated by using Eqs. (9), (10) and (11).

9 $$\Delta {\text{G}}^\circ \, = \, - {\text{RT ln}}\, k_{c}$$

10 $$kc = \frac{{C}_{Ae}}{{C}_{e}}$$

11 $$\mathrm{ln }kc = \frac{\mathrm{\Delta S}^{\_\!\!\!\!\circ} }{R}-\frac{\mathrm{\Delta H}^{\_\!\!\!\!\circ} }{RT}$$

where, *C*_*ae*_ (mg L^−1^) is the equilibrium concentration, C_e_ (mg L^−1^) denotes equilibrium metal ion concentration in the bulk solution, T is the reaction temperature (K) and R is the universal constant (8.314 J mol^−1^ K^−1^). The value of ΔSº and ΔHº were determined using the intercept and slope of the plot between ln *k*_*c*_ versus 1/*T*^129^.

### Kinetics

The comprehension of how metal ions adhere to the exterior of an adsorbent is of utmost significance in the endeavour to formulate efficient wastewater treatment systems^130^. The influence exerted on this process by the attributes of the adsorbent can be assessed with the aid of various models^131^. Presently, our investigation involves the experimentation with diverse models to prognosticate the speed with which heavy metal ions will attach themselves to the surface of a distinct material capable of eliminating them from wastewater.

#### Pseudo-first order kinetics

The pseudo-first-order model refers to how certain substances attach to surfaces. It's a way to understand how quickly this attachment happens, and it's often used in scientific research. The model is described by a mathematical equation, which helps researchers study these processes in more detail (Eq. 12).

12 $$log\left({q}_{e}-{q}_{t}\right)={\text{log}}\left({q}_{e}\right)-\frac{{k}_{s}}{2.303}t$$

where k_s_ is the equilibrium rate constant and calculated from the slope $log({q}_{e}-{q}_{t}$) vs time (t). The *q*_*t*_ and *q*_*e*_ are the adsorption capacities (mg/g) at time *t* and equilibrium, respectively^132^.

#### Pseudo-second order kinetics

The process involves the absorption of a material onto a surface. It is believed that the rate at which this happens is limited by the ability of the material to stick to the surface. This process includes a type of absorption that involves a chemical reaction^133^. The mathematical expression of the pseudo-second-order model is shown in Eq. (13).

13 $$\frac{t}{{q}_{t}}= \frac{1}{{k\mathrm{^{\prime}}}_{2}{q}_{e}}+\frac{1}{{q}_{e}}\mathrm{t }$$

14 $$h= {k^{\prime}}_{2}{q}_{e}^{2}$$

Here, ${k{\prime}}_{2}$ and h are constants that can be calculated from the plot between *t/q*_*t*_ vs *t*.

Recent studies have successfully applied this model to study heavy metal adsorption processes^120,133^.

## Significance of biosorption methods for heavy metal reduction

Biosorption, particularly the utilization of natural or modified biomaterials, presents a promising environmentally friendly technique for the reduction of heavy metals. It presents several benefits, such as the utilization of low-cost adsorbents, achieving high efficiency, and requiring minimal chemical resources. The pseudo-second-order kinetics model, commonly employed in the explanation of biosorption, grants valuable insights into the underlying mechanisms of the process. The comprehension of these mechanisms is of utmost importance to optimize biosorption processes and develop effective treatment systems. In summary, biosorption makes a significant contribution to the present understanding of environmentally friendly approaches to heavy metal reduction, providing sustainable solutions for the remediation of the environment.

## Conclusion and future prospects

Water contamination caused by heavy metals is a significant problem that affects both humans and animals. Heavy metal ions can cause severe health problems such as liver and kidney damage, skin disorders, cognitive impairment and even cancer. To prevent the harmful effects of these toxic metals, it is important to find an eco-friendly and cost-effective method to remove heavy metal ions contamination from wastewater. Biosorption is an eco-friendly method based on the biomass derived from plant, algal, and agricultural waste and microbes. This method is environmentally friendly and does not require much investment. This review provides basic to advanced knowledge to the research about heavy metal contamination and their eco-friendly removal process.

## Data Availability

All data generated or analysed during this study are included in this published article.
